# Supplementary material for: Sex-dependent interactions between prodromal intestinal inflammation and LRRK2 G2019S in mice promote endophenotypes of Parkinson’s disease
Source: Commun Biol. 2024 May 15;7:570. doi: 10.1038/s42003-024-06256-9 (PMC11096388; doi:10.1038/s42003-024-06256-9)
Supplement: Supplementary file 1 — Supplementary Information [file 42003_2024_6256_MOESM1_ESM.pdf]

Supplementary Figure 1

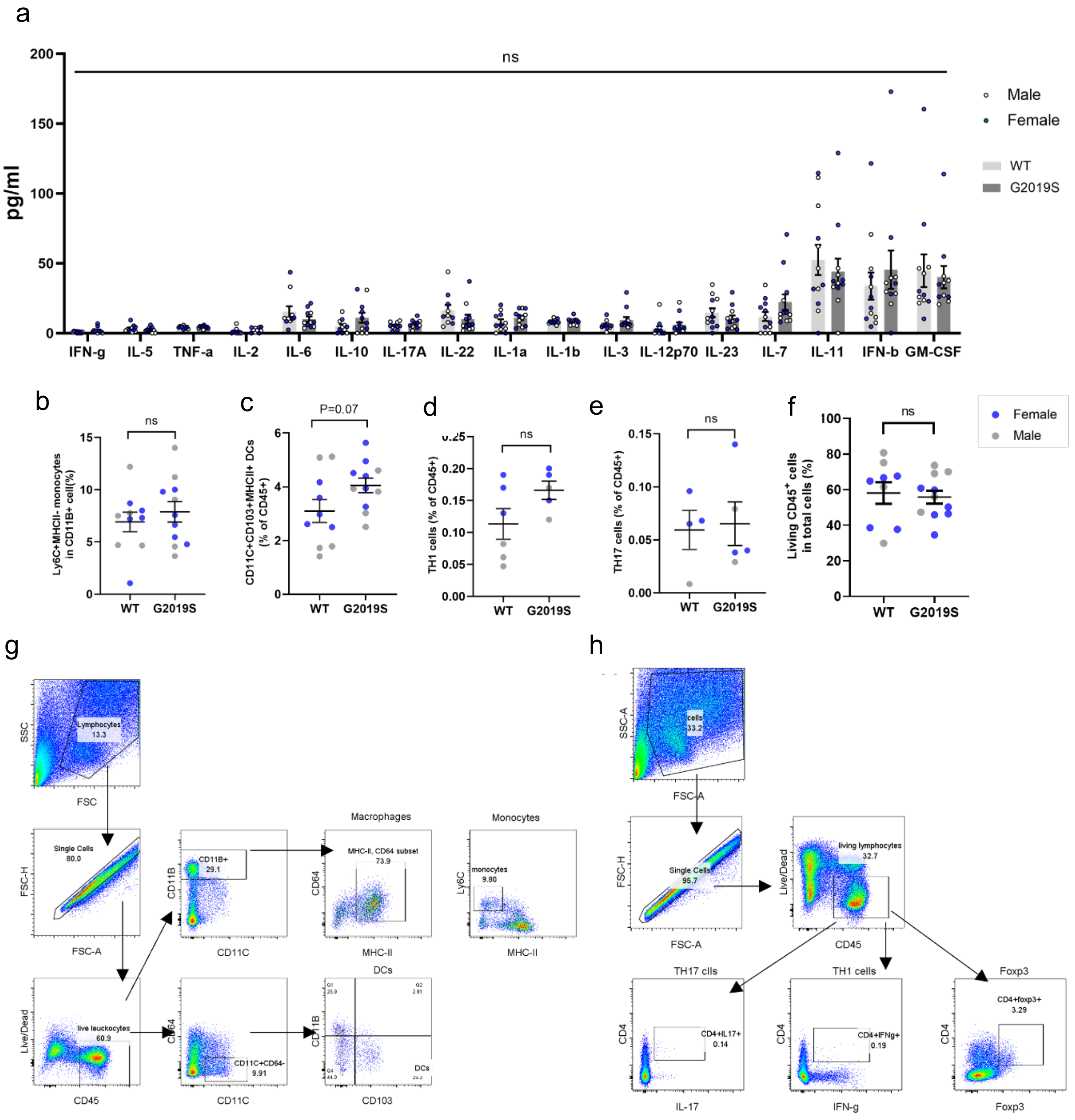

Fig. S1: Serum cytokines and colonic lamina proprial immune cell subsets in response to DSS-induced intestinal inflammation in hLRRK2^{G2019S} Tg mice. (**a**) Serum cytokines in hLRRK2G2019S Tg mice (G2019S) relative to wildtype (WT) littermate controls on day 7 of DSS treatment (two-tailed t-test, n=12). (**b**) Monocytes, (c) dendritic cells, (**d**) TH1 cells and (**e**) TH17 (**f**) CD45+ cells from the colonic lamina propria of G2019S vs. WT mice on day 7 of DSS treatment (two-tailed t-test, (b)-(c), WT: n=8, G2019S: n=11; (d)-(e), WT: n=6, G2019S: n=5). Data are representative of two independent experiments. Data are represented as mean ±SEM; ns= not statistically significant.

Supplementary Figure 2

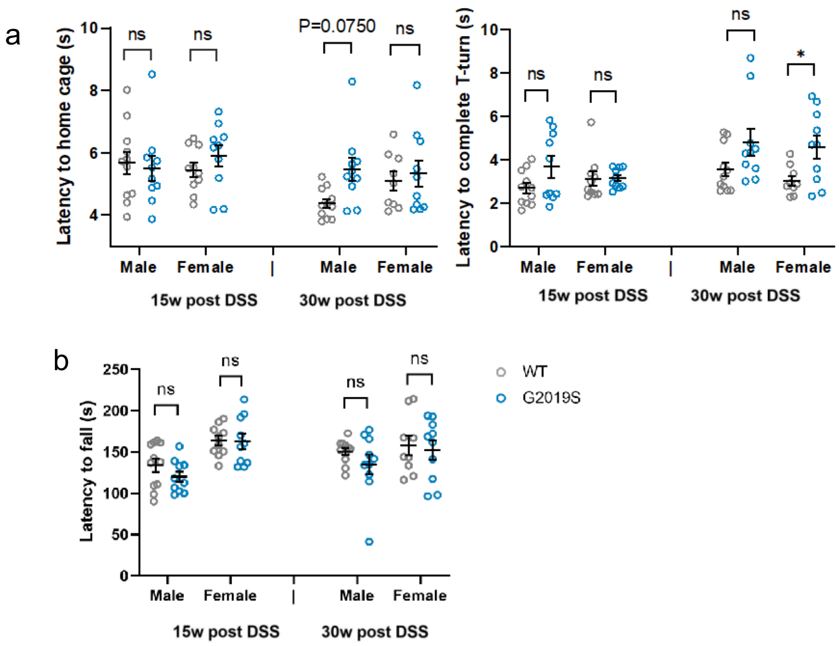

Fig. S2: Behavioral performance in additional motor tasks for hLRRK2^{G2019S} Tg mice after prodromal DSS-induced intestinal inflammation. (**a**) Pole descent test: latency to return to the home cage (left) and latency to complete T turn on the pole (right). (**b**) Rotarod test for hLRRK2^{G2019S} Tg mice (G2019S) compared to wildtype (WT) littermate controls at 15 and 30 weeks post DSS treatment (Two-way ANOVA with Sidak, n=10). Data are representative of three independent experiments. Data are presented as mean ±SEM, **P*<0.05.

Supplementary Figure 3

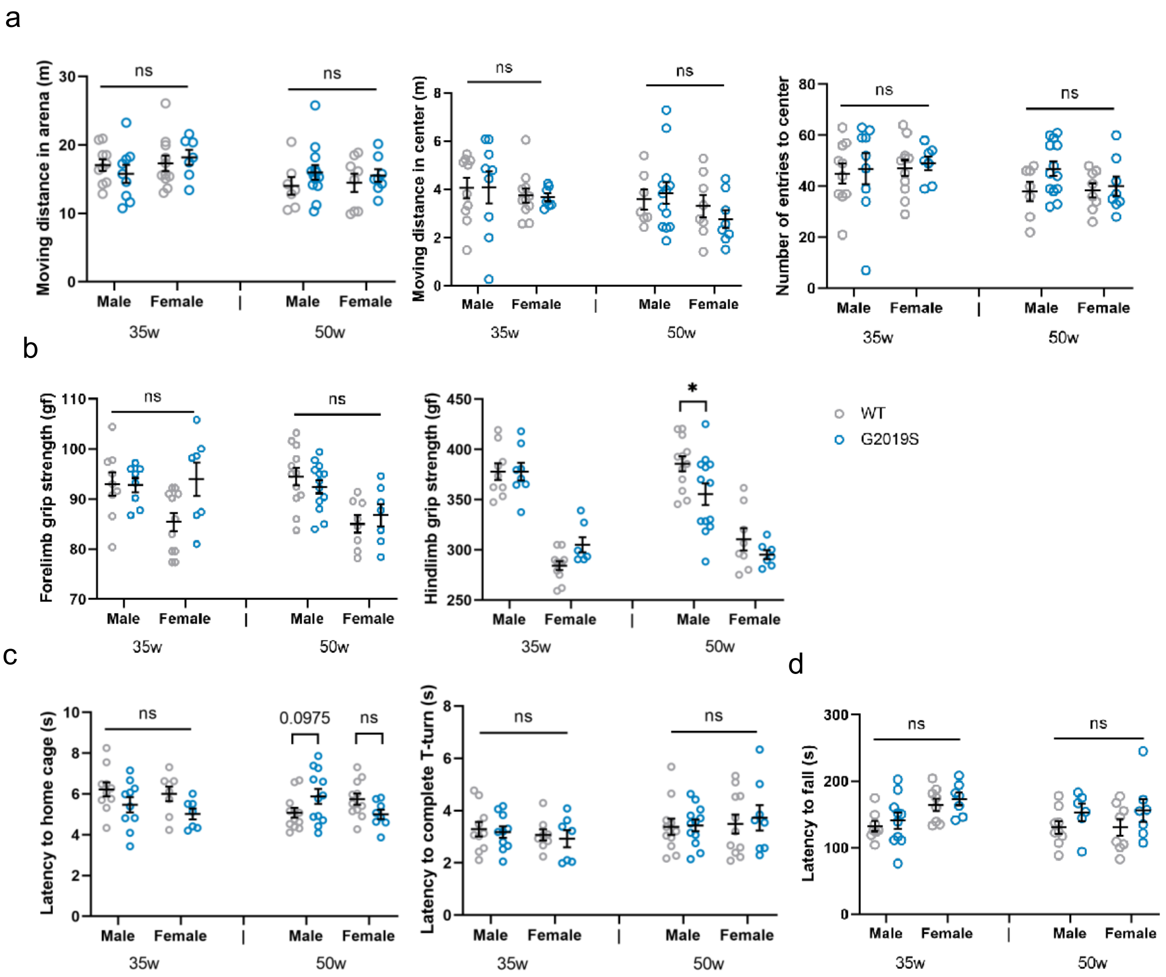

Fig. S3: Vehicle-treated (water; untreated) hLRRK2^{G2019S} Tg mice and WT littermate controls in behavioral tests for motor ability. (**a**) Open field test: moving distance in the arena (left), center (middle), and number of entries to the center (right) after 10 min of exploration. WT_male and female (35w): n=10, G2019S male (35w and 50w): n=10, G2019S female (35w and 50w): n=7, WT male (50w): n=7, WT female (50w): n=8. (**b**) Grip strength for forelimbs (left) and hindlimbs (right). N=7-13 (**c**) Pole descent test: latency to return to the home cage (left) and latency to complete T turn on the pole (right). (**d**) Rotarod test for hLRRK2^{G2019S} Tg mice and WT littermate controls at 35 and 50 weeks of age (two-way ANOVA with Sidak; n=7-11). Data are representative of three independent experiments. Data are presented as mean ±SEM, *P<0.05.

Supplementary Figure 4

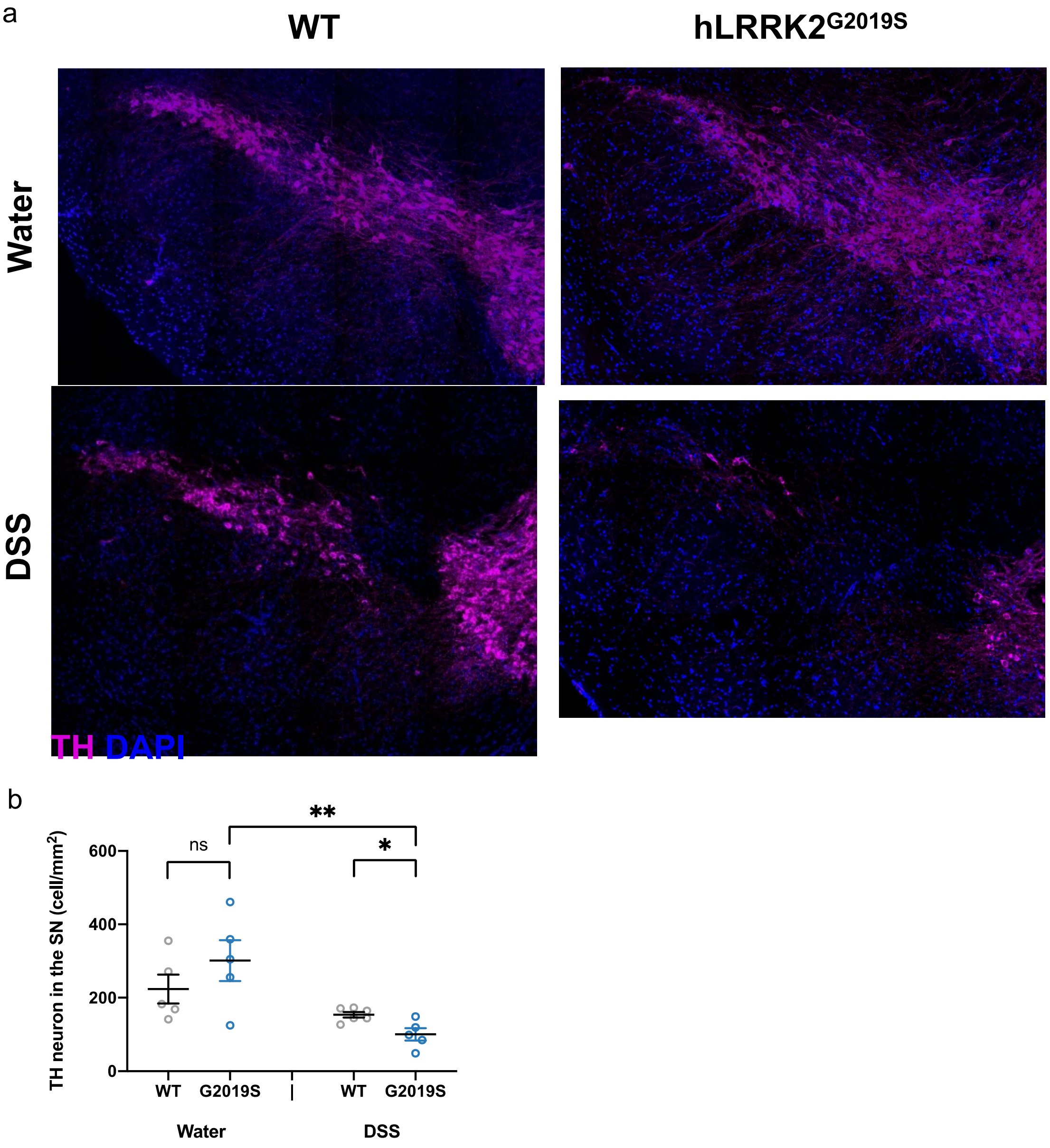

Fig. S4: TH neurons in the SN of male hLRRK2^{G2019S} Tg mice after Vehicle (Water) or DSS treatment. (**a**) Representative images of TH^{+} neurons (magenta) in the SN of male hLRRK2^{G2019S} Tg mice and WT littermates at 30 weeks post treatment with DSS or water as a negative control (52 weeks of age). (**b**) Number of TH^{+} neurons in the SN of male hLRRK2^{G2019S} Tg mice and WT littermates at 32 weeks post treatment with DSS or water as a negative control (52 weeks of age) (two-way ANOVA with Sidak; WT (water), G2019S (water) and G2019S (DSS): n=5, WT (DSS): n=6). Data are representative of two independent experiments. Data for the DSS groups are as in Figure 2G. Data are presented as means ±SEM, **P*<0.05, *****P*<0.001.

Supplementary Figure 5

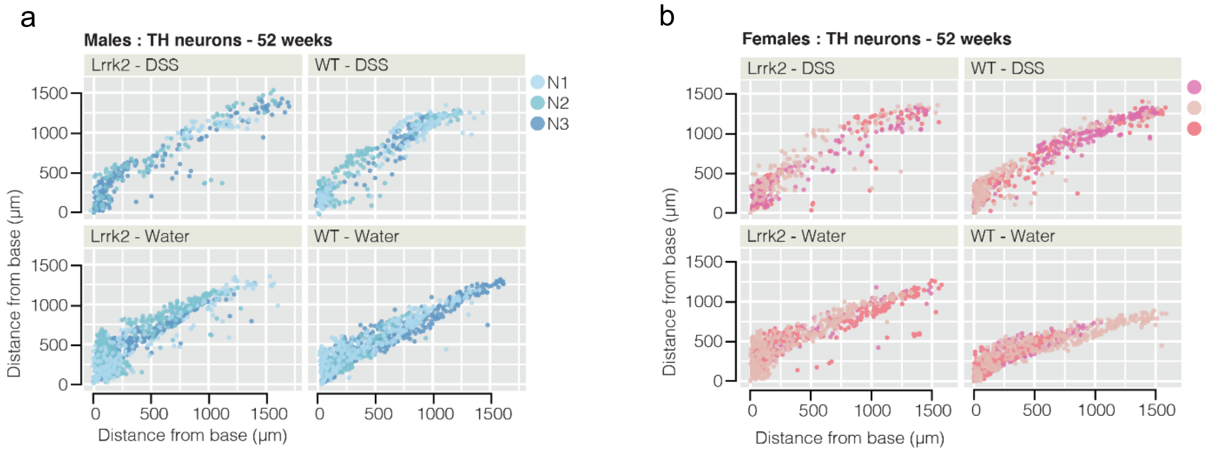

Fig. S5: Projection of TH neurons in the SN in a 2D map. TH neurons in the SN of male (**a**) and female (**b**) hLRRK2^{G2019S} Tg mice and WT littermates at 30 weeks post treatment with DSS or water as a negative control (52 weeks of age). Each dot on the map represents a TH neuron quantified on a real image. n=3.

Supplementary Figure 6

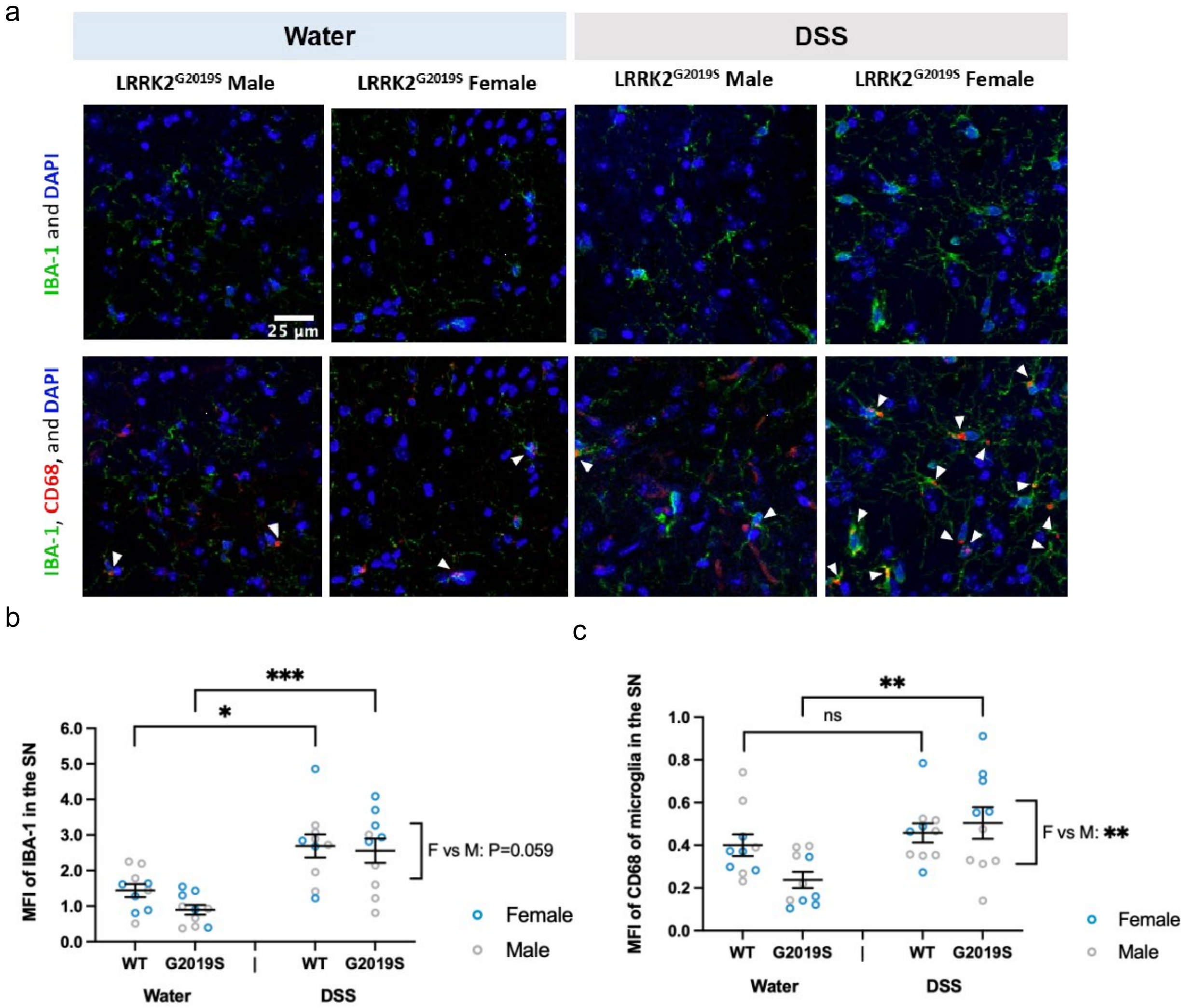

Fig. S6: IBA-1 and CD68 in the SN of hLRRK2^{G2019S} Tg mice after DSS treatment. (**A**) Representative images of IBA-1^{+} microglia (green) and CD68 (red) in SN of male and female hLRRK2^{G2019S} Tg mice and WT littermates at 32 weeks post DSS treatment (52 weeks of age). Arrows point to CD68 as puncta within the IBA-1 signal. (**B**) MFI of IBA-1^{+} microglia in the SN. (**C**) MFI of CD68 within the IBA-1^{+} microglia in the SN. (Two-way ANOVA with Sidak; n=10). Data are representative of two independent experiments. Data are presented as means ±SEM, *P<0.05 , **P<0.01, ***P<0.001 .

Supplementary Figure 7

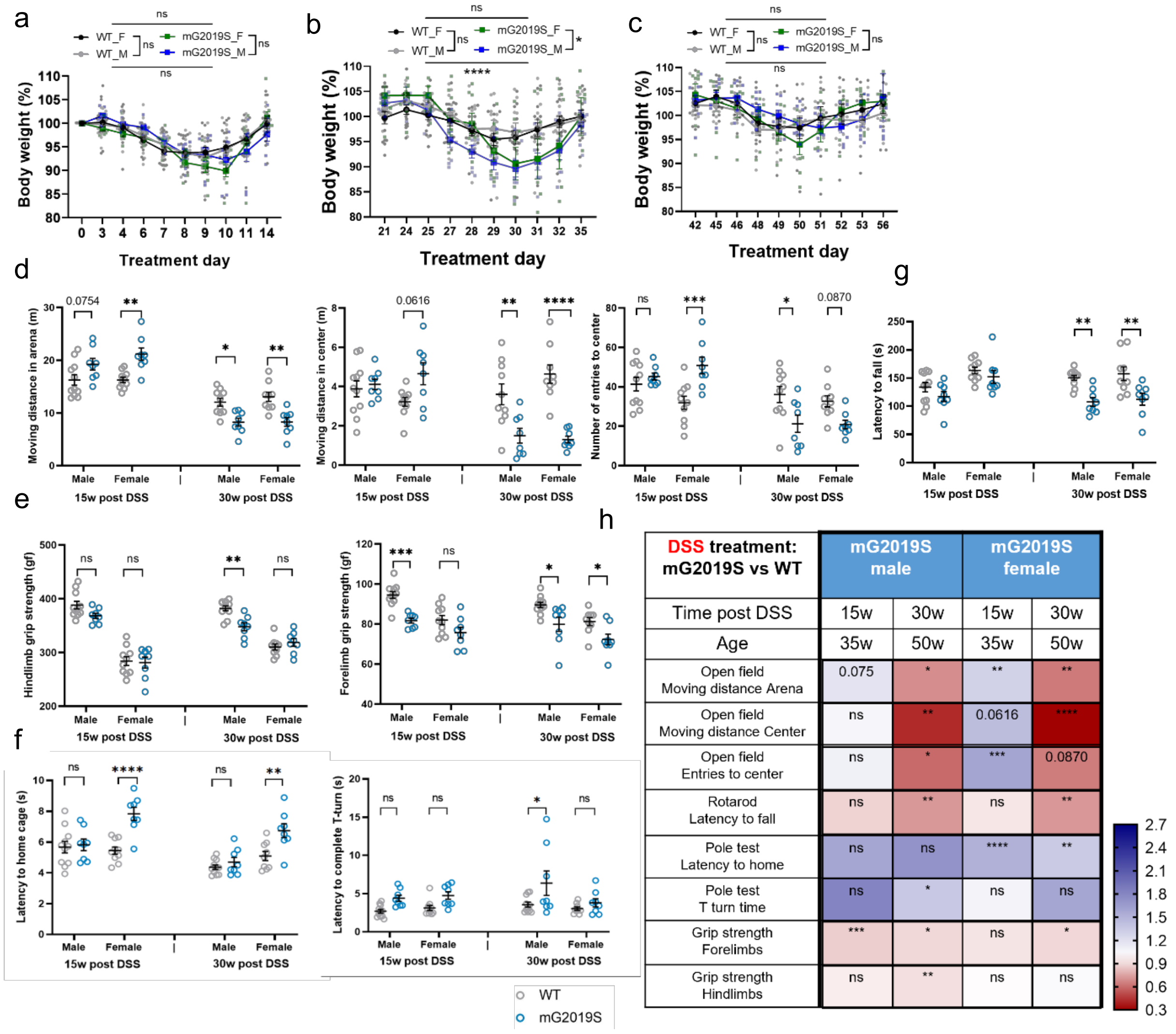

Fig. S7: Body weight loss and motor impairments in mLRRK2^{G2019S} KI mice after early DSS treatment. (**a**)-(**c**) Body weight normalized to starting weight (day 0) for male (M) and female (F) mLRRK2^{G2019S} KI mice (mG2019S) and wildtype littermate controls (WT) over 3 rounds DSS treatment (two-way ANOVA with Sidak, n=8-11). (**d**) Open field test: moving distance in the arena (left), center (middle) and number of entries to the center (right) after 10 min exploration, (**e**) Grip strength for the hindlimbs (left) and forelimbs (right), (**f**) Pole descent test: latency to return to the home cage (left) and latency to complete T turn on the pole (right), (**g**) Rotarod test of mLRRK2^{G2019S} KI mice and wildtype littermates at 15 and 30 weeks post DSS treatment. (**h**) Summary of behavioral testing results for mLRRK2^{G2019S} KI mice (mG2019S) relative to WT littermate controls at 15 and 30 weeks post DSS treatment (two-way ANOVA with Sidak, WT male and female: n=10, mG2019S male and female: n=8). Data are representative of two independent experiments. Data are presented as means ±SEM, *P<0.05, ***P*<0.01, ****P*<0.001 and *****P*<0.0001.

Supplementary Figure 8

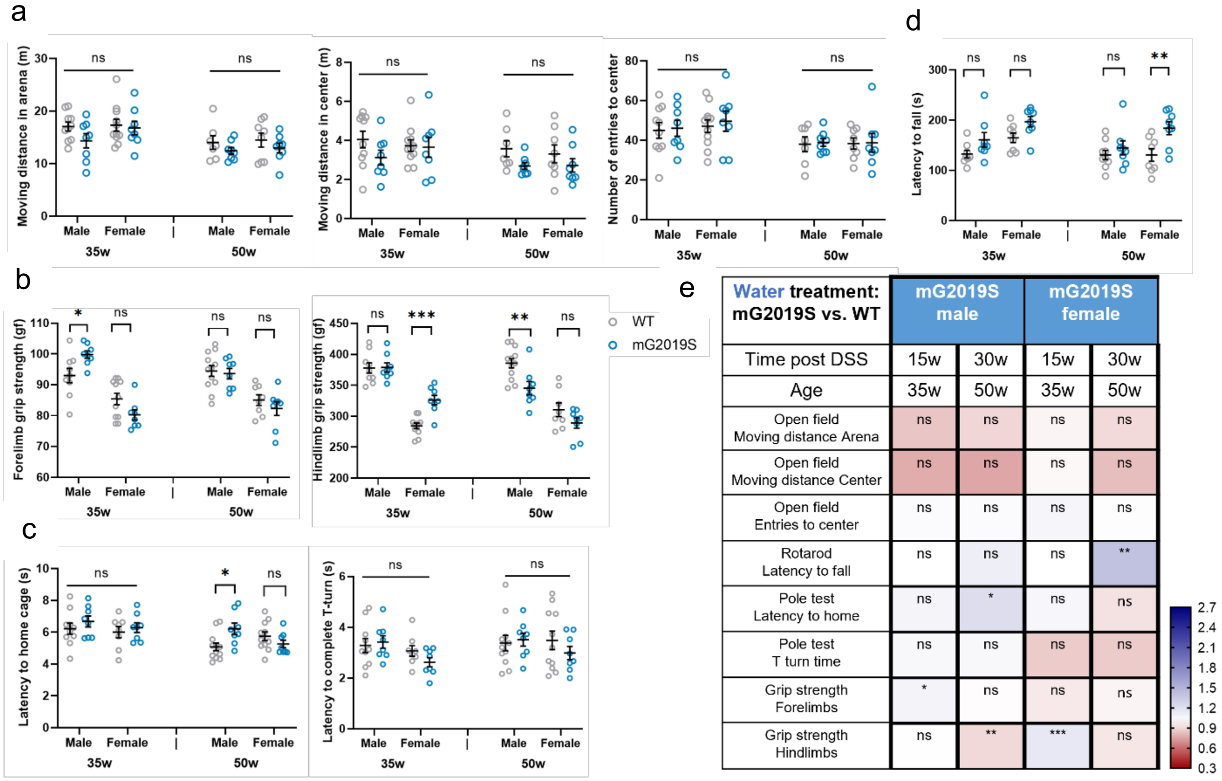

Fig. S8: Vehicle-treated (water; untreated) mLRRK2^{G2019S} KI mice and WT littermate controls in behavioral tests for motor ability. (**a**) Open field test: moving distance in the arena (left), center (middle) and number of entries to the center (right) after 10 min of exploration, (**b**) Grip strength for the forelimbs (left) and hindlimbs (right), (**c**) Pole descent test: latency to return to the home cage (left) and latency to complete T turn on the pole (right), (**d**) Rotarod test for mLRRK2^{G2019S} KI mice (mG2019S) and wildtype (W) littermate controls at 15 and 30 weeks post DSS treatment. (**e**) Summary of behavioral testing results for mLRRK2^{G2019S} KI mice (mG2019S) and WT littermate controls (two-way ANOVA with Sidak; WT male and female: n=10, mG2019S male and female: n=8). Data are representative of two independent experiments. Data are presented as means ±SEM, **P*<0.05, ***P*<0.01 and ****P*<0.001

Supplementary Figure 9

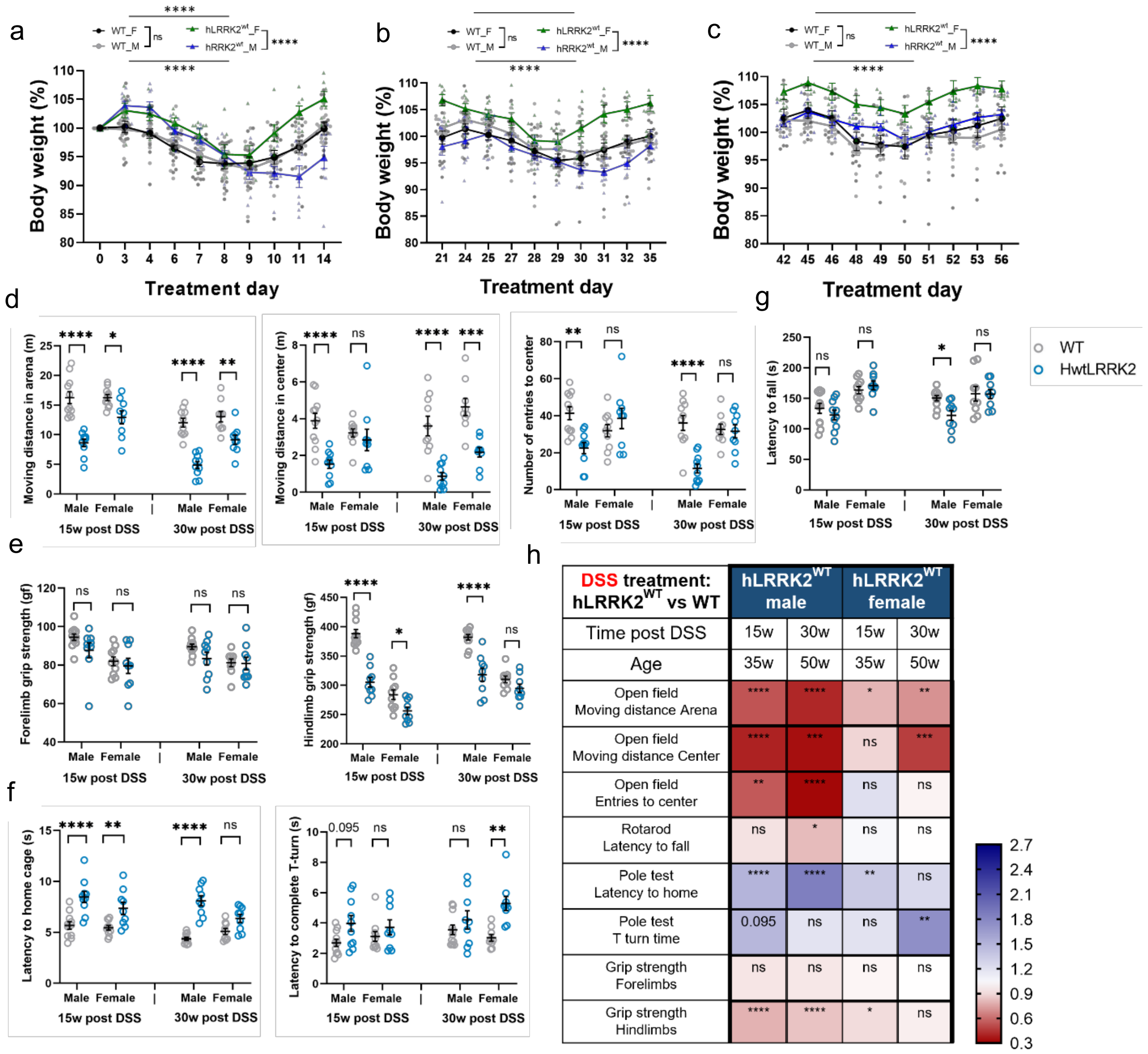

Fig. S9: Body weight loss and motor impairments in hLRRK2^{WT} Tg mice after DSS treatment. (**a**)-(**c**) Body weight normalized to starting weight (day 0) for male (M) and female (F) hLRRK2^{WT} Tg mice and wildtype littermates (WT) during each of the 3 rounds of DSS treatment. (**d**) Open field test: moving distance in the arena (left), center (middle) and number of entries to the center (right) after 10 min of exploration, (**e**) Grip strength for the forelimbs (left) and hindlimbs (right), (**f**) Pole descent test: latency to return to the home cage (left) and latency to complete T turn on the pole (right), (**g**) Rotarod test of hLRRK2^{WT} Tg mice and wildtype (WT) littermate controls at 15 and 30 weeks post DSS treatment. (**h**) Summary of behavioral testing results for hLRRK2^{WT} Tg mice and WT controls at 15 and 30 weeks post DSS treatment (two-way ANOVA with Sidak, n=10). Data are representative of two independent experiments. Data are presented as mean ±SEM, **P*<0.05, ***P*<0.01, ****P*<0.001 and *****P*<0.0001.

Supplementary Figure 10

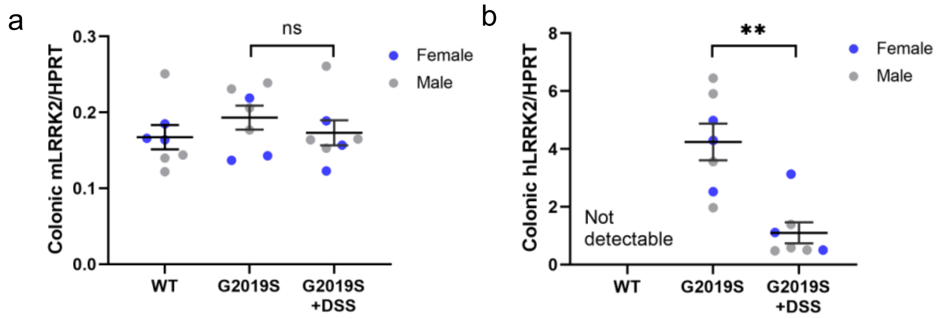

Fig. S10: Colonic expression of *LRRK2* in the hLRRK2^{G2019S} Tg mice at baseline and after 7 days of DSS treatment. mRNA levels of endogenous mouse *LRRK2* (**a**) and human *LRRK2 G2019S* transgene (**b**) were examined by real time quantitative PCR in the colon from male and female hLRRK2^{G2019S} Tg mice (G2019S) and wildtype littermates (WT), at baseline and 7 days after DSS treatment (two-way ANOVA with Sidak, n=7). Gene expression is normalized to expression of *HPRT*. Data are representative of three independent experiments. Data are presented as mean ±SEM, ***P*<0.01.

Supplementary Figure 11

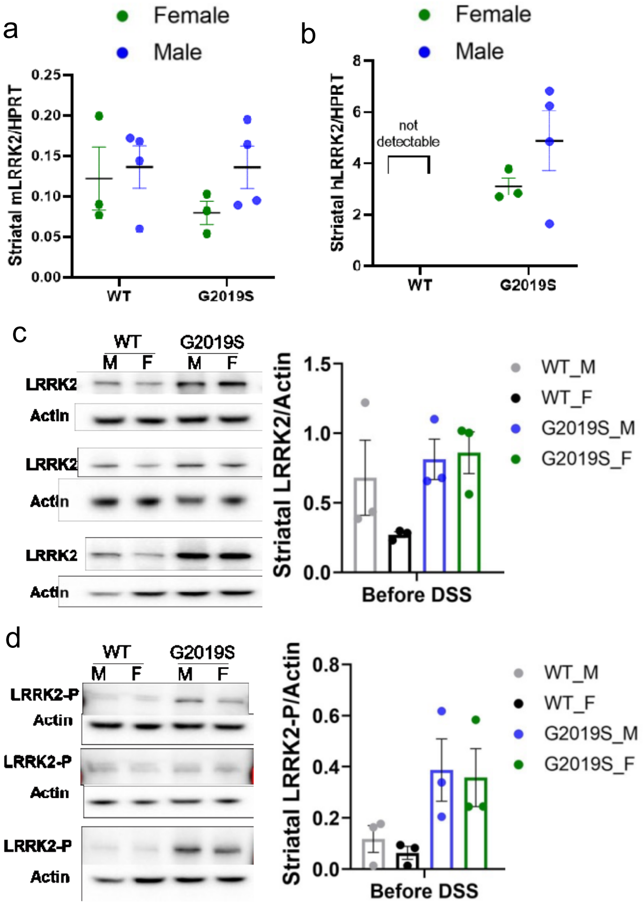

Fig. S11: Striatal expression of LRRK2 in hLRRK2^{G2019S} Tg mice. mRNA levels of endogenous mouse *LRRK2* (**a**) and human *LRRK2 G2019S* transgene (**b**) in the striatum from male and female hLRRK2^{G2019S} Tg mice (G2019S) and wildtype littermate controls (WT). (**c**) Total LRRK2 and (**d**) phosphorylated LRRK2 (both endogenous mouse and human transgenic LRRK2) in the striatum from LRRK2^{G2019S} Tg mice and WT controls. Data are presented as mean ±SEM.

Supplementary Figure 12

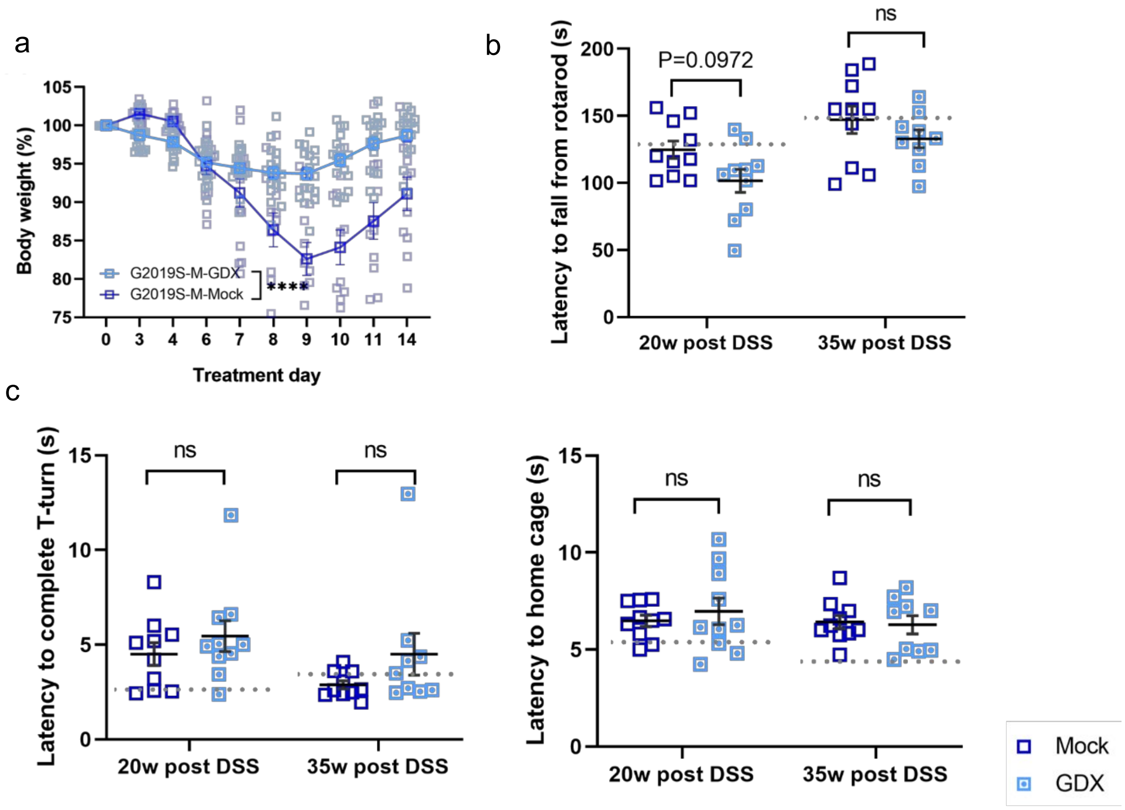

Fig. S12: Body weight loss and behavioral performance in additional motor tasks for gonadectomized (GDX) male hLRRK2^{G2019S} Tg mice after DSS treatment. (**a**) Body weight normalized to starting weight (Day 0) for gonadectomized (GDX) or Mock surgery-exposed (Mock) male hLRRK2^{G2019S} Tg mice (G2019S-M) during the first round of DSS treatment. (**b**) Rotarod test and (**c**) Pole descent test: latency to return to the home cage (left) and latency to complete T turn on the pole (right) at 20 and 35 weeks post DSS treatment (two-way ANOVA with Sidak, mock and GDX (20w post DSS): n=10, GDX (35w post DSS): n=9). Data are representative of two independent experiments. Data are presented as mean ±SEM, ****P<0.0001. Dotted lines indicate the mean value for WT littermates as a reference.

Supplementary Figure 13

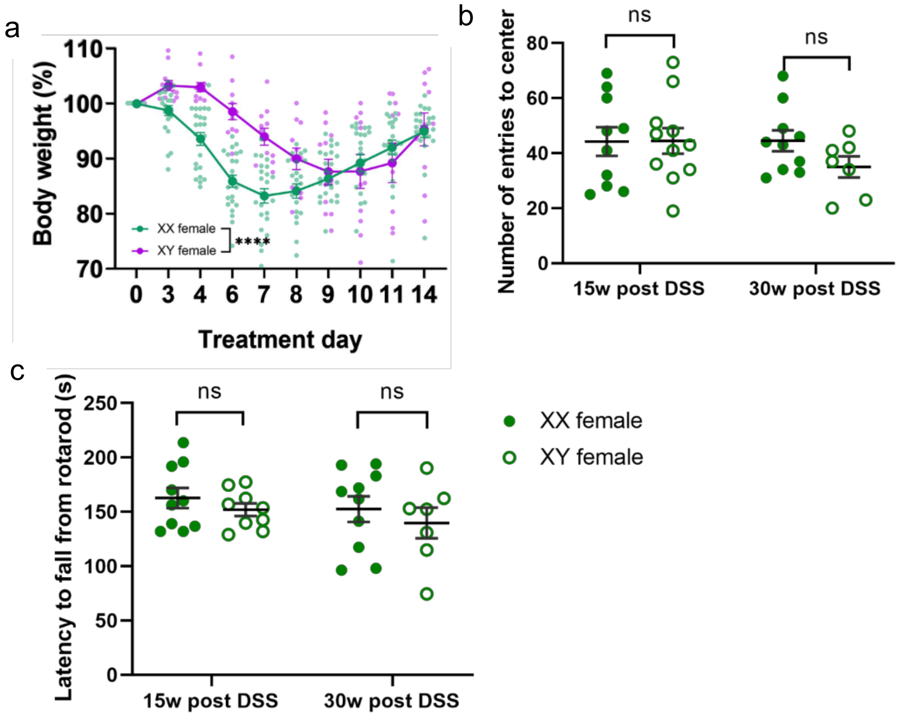

Fig. S13: Body weight loss and behavioral performance in additional motor tasks for XY female hLRRK2^{G2019S} Tg mice after DSS treatment. (**a**) Body weight normalized to starting weight (day 0) for XX and XY female hLRRK2^{G2019S} Tg mice during the first round of DSS treatment. (**b**) Open field test: number of entries to the center after 10 min of exploration. (**c**) Rotarod test for XX and XY female hLRRK2^{G2019S} Tg at 15 and 30 weeks post DSS treatment (two-way ANOVA with Sidak, XX female n=10, XY female (15w post DSS): n=9, XY female (30w post DSS: n=7). Data are representative of two independent experiments. Data are presented as mean ±SEM, ****P<0.0001

Supplementary Figure 14

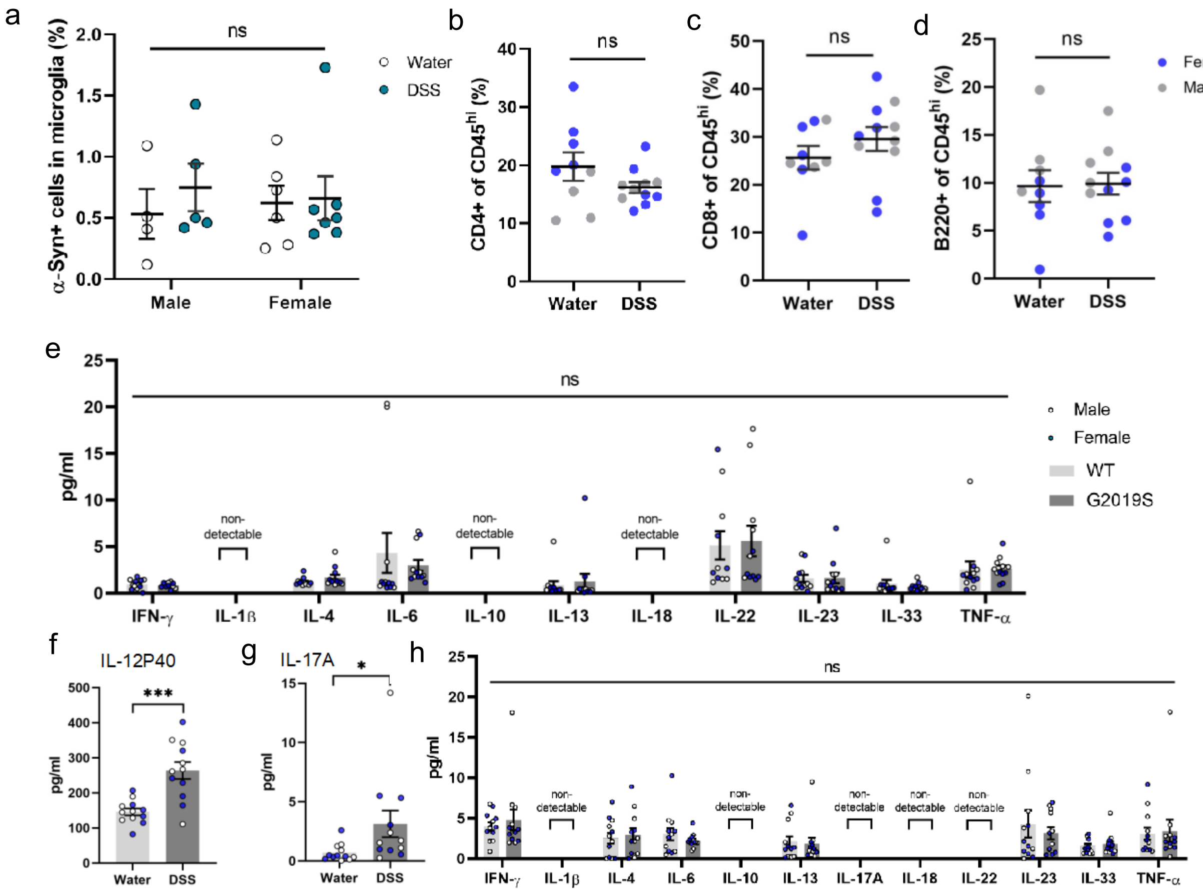

Fig. S14: DSS-induced alterations in brain immune profiles of hLRRK2^{G2019S} Tg mice relative to WT littermate controls, prior to the onset of neuropathological and behavioral endophenotypes of PD. Flow cytometry of brain parenchymal cells for (**a**) a-Syn^{+} microglia (two-way ANOVA with Sidak, G2019S male (water): n=4, G2019S (DSS): n=5, G2019S female: n=6), (**b**) CD4 T cells, (**c**) CD8 T cells and (**d**) B cells from hLRRK2^{G2019S} Tg mice at 1 week after the last round of treatment with DSS or vehicle (water) (paired t-test, G2019S (water): n=9, G2019S (DSS): n=12). (**e**) Serum cytokine concentrations for (**f**) IL-12P40 and (**g**) IL-17A, and (**h**) various cytokines in CSF from hLRRK2^{G2019S} Tg mice at 1 week after the last round of treatment with DSS or vehicle (paired t-test, G2019S (water): n=9, G2019S (DSS): n=12). Data are representative of two independent experiments. Data are presented as mean ±SEM, *P<0.05 and ***P<0.001.

Supplementary Figure 15

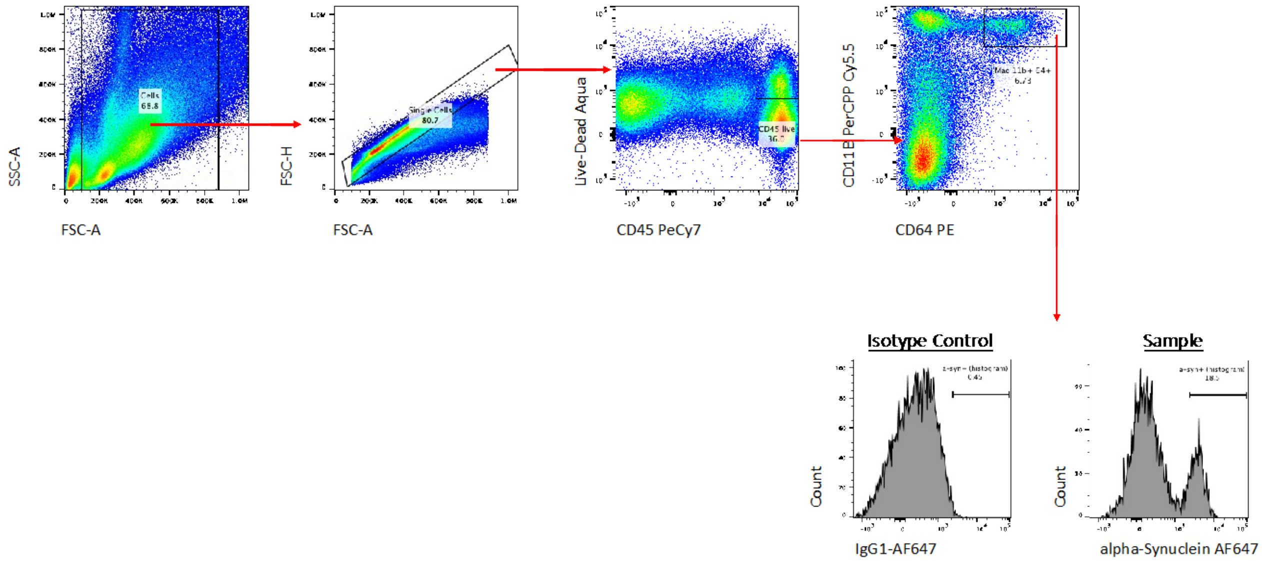

Figure S15. Gating strategy for α-synuclein+ macrophages from the colonic lamina propria
