# Supplementary material for: Sex-dependent interactions between prodromal intestinal inflammation and LRRK2 G2019S in mice promote endophenotypes of Parkinson’s disease
Source: Commun Biol. 2024 May 15;7:570. doi: 10.1038/s42003-024-06256-9 (PMC11096388; doi:10.1038/s42003-024-06256-9)
Supplement: Supplementary file 2 — Description of Supplementary Materials [file 42003_2024_6256_MOESM2_ESM.docx]

**Description of Additional Supplementary Files**

**File name:** Supplementary Data 1

**Description:** The source data for Figure 1

**File name:** Supplementary Data 2

**Description:** The source data for Figure 2

**File name:** Supplementary Data 3

**Description:** The source data for Figure 3

**File name:** Supplementary Data 4

**Description:** The source data for Figure 4

**File name:** Supplementary Data 5

**Description:** The source data for all Supplementary Figures
