# Supplementary material for: Sex-dependent interactions between prodromal intestinal inflammation and LRRK2 G2019S in mice promote endophenotypes of Parkinson’s disease
Source: Commun Biol. 2024 May 15;7:570. doi: 10.1038/s42003-024-06256-9 (PMC11096388; doi:10.1038/s42003-024-06256-9)
Supplement: Supplementary file 8 — Reporting Summary [file 42003_2024_6256_MOESM8_ESM.pdf]

nature portfolio

Corresponding author(s): DBPR NCOMMS-23-1104

Last updated by author(s): Apr 17, 2024

Reporting Summary

Nature Portfolio wishes to improve the reproducibility of the work that we publish. This form provides structure for consistency and transparency in reporting. For further information on Nature Portfolio policies, see our Editorial Policies and the Editorial Policy Checklist.

Statistics

For all statistical analyses, confirm that the following items are present in the figure legend, table legend, main text, or Methods section.

n/a	Confirmed	
☐	☒	The exact sample size (*n*) for each experimental group/condition, given as a discrete number and unit of measurement
☐	☒	A statement on whether measurements were taken from distinct samples or whether the same sample was measured repeatedly
☐	☒	The statistical test(s) used AND whether they are one- or two-sided<br>*Only common tests should be described solely by name; describe more complex techniques in the Methods section.*
☒	☐	A description of all covariates tested
☐	☒	A description of any assumptions or corrections, such as tests of normality and adjustment for multiple comparisons
☐	☒	A full description of the statistical parameters including central tendency (e.g. means) or other basic estimates (e.g. regression coefficient) AND variation (e.g. standard deviation) or associated estimates of uncertainty (e.g. confidence intervals)
☒	☐	For null hypothesis testing, the test statistic (e.g. *F*, *t*, *r*) with confidence intervals, effect sizes, degrees of freedom and *P* value noted<br>*Give P values as exact values whenever suitable.*
☒	☐	For Bayesian analysis, information on the choice of priors and Markov chain Monte Carlo settings
☒	☐	For hierarchical and complex designs, identification of the appropriate level for tests and full reporting of outcomes
☒	☐	Estimates of effect sizes (e.g. Cohen's *d*, Pearson's *r*), indicating how they were calculated

Our web collection on statistics for biologists contains articles on many of the points above.

Software and code

Policy information about availability of computer code

Data collection	Zen Black 2.3<br>BD FACSDiva<br>CleverSys TopScan<br>Microsoft Excel 365
Data analysis	Fiji-Windows 64-bit<br>FlowJo 10<br>Graphpad Prism 8.0

For manuscripts utilizing custom algorithms or software that are central to the research but not yet described in published literature, software must be made available to editors and reviewers. We strongly encourage code deposition in a community repository (e.g. GitHub). See the Nature Portfolio guidelines for submitting code & software for further information.

Data

Policy information about availability of data

All manuscripts must include a data availability statement. This statement should provide the following information, where applicable:

- Accession codes, unique identifiers, or web links for publicly available datasets
- A description of any restrictions on data availability
- For clinical datasets or third party data, please ensure that the statement adheres to our policy

The datasets generated during and/or analysed during the current study are available from the corresponding author on reasonable request.

Human research participants

Policy information about studies involving human research participants and Sex and Gender in Research.

Reporting on sex and gender	No human research data was collected for this study
Population characteristics	See above
Recruitment	See above
Ethics oversight	See above

Note that full information on the approval of the study protocol must also be provided in the manuscript.

Field-specific reporting

Please select the one below that is the best fit for your research. If you are not sure, read the appropriate sections before making your selection.

☒ Life sciences ☐ Behavioural & social sciences ☐ Ecological, evolutionary & environmental sciences

For a reference copy of the document with all sections, see nature.com/documents/nr-reporting-summary-flat.pdf

Life sciences study design

All studies must disclose on these points even when the disclosure is negative.

Sample size	Sample size of all the experiments are described in figure legends, and the sample size were sufficient to conduct reasonable statistical analyses where applicable. Sample size was determined using power calculation allowing the use of statistical analysis. Statistical significance of differences between experimental groups and controls was assessed by two-tailed unpaired t-test, and one or two way ANOVA, as indicated in the legends. In all cases, p values < 0.05 were considered as statistically significant.
Data exclusions	No data were excluded from analysis
Replication	Results of animal behavioral tests, in vivo experiments and ex vivo experiments were replicated with 2-3 batches of mice to verify the results<br>Results of molecular biology experiments were replicated at least once to verify the results
Randomization	Samples are allocated to experimental groups randomly
Blinding	Investigators are blinded to the experimental groups during data collection. The mice have ID only on the animal card but not description of treatments or genetic background on the animal card. Therefore, investigators can only distinguish the mouse ID but not groups during data collection.

Reporting for specific materials, systems and methods

We require information from authors about some types of materials, experimental systems and methods used in many studies. Here, indicate whether each material, system or method listed is relevant to your study. If you are not sure if a list item applies to your research, read the appropriate section before selecting a response.

Materials & experimental systems

n/a	Involved in the study
☐	☒ Antibodies
☒	☐ Eukaryotic cell lines
☒	☐ Palaeontology and archaeology
☐	☒ Animals and other organisms
☒	☐ Clinical data
☒	☐ Dual use research of concern

Methods

n/a	Involved in the study
☒	☐ ChIP-seq
☐	☒ Flow cytometry
☒	☐ MRI-based neuroimaging

Antibodies

Antibodies used

Ly6G-BV421(Biolegend, cat# 127628 1:200 clone: 1A8),
Ly6C-BV605 (Biolegend, cat# 128036 1:200 clone: HK1.4),
CD4-BV650 (Biolegend, cat# 100469 1:200 clone: GK1.5),
NK1.1-BV711 (Biolegend, cat# 108745 1:200 clone: PK136),
B220-FITC (Biolgend, cat# 103205 1:200 clone:RA3-6B2),
CD8a-PerCP-Cy5.5(Tonbo bioscience, cart# 65-0081-U025 1:200 clone:53-6.7),
CD45-PE-Cy7 (Tonbo bioscience, cat# 60-0451-U100 1:200 clone:30-F11),
CD11c-APC-eFlour780 (Thermofisher, cat# 47-0114-82 1:200 clone:N418),
CD3-AF700 (Biolegend, cat# 100215 1:200 clone:),
CD11b-PE-eFlour610 (Thermofisher, cat# 61-0112-80 1:200 clone:M1/70),
MHCII-FITC (Thermofisher, cat# 11-5321-82 1:200 clone:),
CD64-PE-Cy7 (Biolegend, cat# 139313 1:200 clone:FcγRI),
IL-17A- FITC (Thermofisher, Cat#: 11-7177-81 1:200 clone:eBio17B7)
IFNg-PE (Thermofisher, Cat# 12-7311-81 1:200 clone: XMG1.2),
TNFa-BV421 (Biolegend, Cat# 506327 1:200 clone:MP6-XT22),
IL-1b-PE (Thermofisher, Cat#12-7114-82 1:200 clone:NJTEN3),
α-synuclein-APC (Novus Biologicals, Cat# NBP1-05194APC 1:100 clone:2A7).
IBA-1: Fujifilm Wako Chemicals, 019-1974, Rabbit anti-mouse, 1:1000 clone: Polyclonal.
IBA-1: Fujifilm Wako Chemicals, 011-27991, Goat anti-mouse, 1:1000 clone: Polyclonal.
CD68 Bio-Rad, MCA1957T, Rat anti-mouse, 1:400 clone: FA-11.
α-synuclein pSer129: Novus Biologicals, NBP2-61121 1:400 clone:Polyclonal .

Secondary antibodies used:
Alexa Fluor 647 AffiniPure Donkey Anti-Chicken (Jackson ImmunoResearch ,703-605-155) 1:1000.
Alexa Fluor 488 AffiniPure Donkey Anti-Rabbit (711-545-152) 1:1000.
Alexa Fluor 594 AffiniPure Donkey Anti-Rat (Jackson ImmunoResearch, 712-585-153) 1:1000.
Alexa Fluor® 647 AffiniPure Donkey Anti-Goat (Jackson ImmunoResearch, 705-605-003) 1:1000.

Validation

Ly6G-BV421(target species: mouse, application: Flow cytometry), validation: https://www.biolegend.com/en-us/products/brilliant-violet-421-anti-mouse-ly-6g-antibody-7161
Ly6C-BV605 (target species: mouse, application: Flow cytometry), validation:https://www.biolegend.com/en-us/products/brilliant-violet-605-anti-mouse-ly-6c-antibody-8727
CD4-BV650 (target species: mouse, application: Flow cytometry), validation: https://www.biolegend.com/en-us/products/brilliant-violet-650-anti-mouse-cd4-antibody-16780
NK1.1-BV711 (target species: mouse, application: Flow cytometry), validation:https://www.biolegend.com/en-us/products/brilliant-violet-711-anti-mouse-nk-1-1-antibody-9576
B220-FITC (target species: mouse, application: Flow cytometry), validation: https://www.biolegend.com/en-us/products/fitc-anti-mouse-human-cd45r-b220-antibody-445
CD8a-PerCP-Cy5.5(target species: mouse, application: Flow cytometry), validation: https://cytekbio.com/products/percp-cyanine5-5-anti-mouse-cd8a-53-6-7?variant=40581194711076
CD11c-APC-eFlour780 (target species: mouse, application: Flow cytometry), validation:https://www.thermofisher.com/antibody/product/CD11c-Antibody-clone-N418-Monoclonal/47-0114-82
CD11b-PE-eFlour610 (target species: mouse, application: Flow cytometry), validation: https://www.thermofisher.com/antibody/product/CD11b-Antibody-clone-M1-70-Monoclonal/61-0112-80
MHCII-FITC (target species: mouse, application: Flow cytometry), validation: https://www.thermofisher.com/antibody/product/MHC-Class-II-I-A-I-E-Antibody-clone-M5-114-15-2-Monoclonal/11-5321-82
CD64-PE-Cy7 (target species: mouse, application: Flow cytometry), validation: https://www.biolegend.com/en-us/products/pe-cyanine7-anti-mouse-cd64-fcgammari-antibody-10062
IL-17A- FITC (target species: mouse, application: Flow cytometry), validation: https://www.thermofisher.com/antibody/product/IL-17A-Antibody-clone-eBio17B7-Monoclonal/11-7177-81
IFNg-PE (target species: mouse, application: Flow cytometry), validation:https://www.thermofisher.com/antibody/product/IFN-gamma-Antibody-clone-XMG1-2-Monoclonal/12-7311-81
TNFa-BV421 (target species: mouse, application: Flow cytometry), validation: https://www.biolegend.com/en-us/products/brilliant-violet-421-anti-mouse-tnf-alpha-antibody-7336
IL-1b-PE (target species: mouse, application: Flow cytometry), validation:https://www.thermofisher.com/antibody/product/IL-1-beta-Pro-form-Antibody-clone-NJTEN3-Monoclonal/12-7114-82
α-synuclein-APC (target species: mouse, application: Flow cytometry), validation: https://www.novusbio.com/products/alpha-synuclein-antibody-2a7_nbp1-05194apc
IBA-1: Fujifilm Wako Chemicals, 019-1974, Rabbit anti-mouse, 1:1000 target species: mouse, application: immunofluorescence),

validation:https://labchem-wako.fujifilm.com/us/product/detail/W01W0101-1974.html
IBA-1: Fujifilm Wako Chemicals, 011-27991, Goat anti-mouse, 1:1000 clone target species: mouse, application: immunofluorescence
CD68, target species: mouse, application: immunofluorescence, validation: https://labchem-wako.fujifilm.com/us/product/detail/W01W0101-2799.html
α-synuclein pSer129: Novus Biologicals, NBP2-61121, target species: mouse, application: Western Blot validation: https://www.novusbio.com/products/alpha-synuclein-antibody_nbp2-61121

Secondary antibodies used:
Alexa Fluor 647 AffiniPure Donkey Anti-Chicken (Jackson ImmunoResearch ,703-605-155) 1:1000. application: immunofluorescence, validation:https://www.jacksonimmuno.com/catalog/products/703-605-155
Alexa Fluor 488 AffiniPure Donkey Anti-Rabbit (711-545-152) 1:1000. application: immunofluorescence, validation: https://www.jacksonimmuno.com/catalog/products/711-545-152
Alexa Fluor 594 AffiniPure Donkey Anti-Rat (Jackson ImmunoResearch, 712-585-153) 1:1000. application: immunofluorescence, validation: https://www.jacksonimmuno.com/catalog/products/712-585-153
Alexa Fluor® 647 AffiniPure Donkey Anti-Goat (Jackson ImmunoResearch, 705-605-003) 1:1000. application: immunofluorescence validation: https://www.jacksonimmuno.com/catalog/products/705-605-003

Animals and other research organisms

Policy information about studies involving animals; ARRIVE guidelines recommended for reporting animal research, and Sex and Gender in Research

Laboratory animals	C57BL/6 mice, 9 weeks old, male and female<br>hLRRK2G2019S Tg mice: C57BL/6J-Tg(LRRK2*G2019S)2AMjff/J, male and female<br>hLRRK2WT Tg mice: B6.FVB-Tg(LRRK2)WT1Mjfa/J, male and female<br>mLRRK2G2019S KI mice: B6.Cg-Lrrk2tm1.1Hlme/J, male and female<br>XY–(Sry+) male mice, fathers of the "Four Core Genotypes" model: B6.Cg-Tg(Sry)2Ei Srydl1Rlb/ArnoJ
Wild animals	No wild animals was involved in this study
Reporting on sex	Both male and females are included in this study.
Field-collected samples	Study didn't involve the samples collected in the field
Ethics oversight	All experiments were performed in accordance with the NIH Guide for the Care and Use of Laboratory Animals using protocols approved by the Institutional Animal Care and Use Committee at UCLA

Note that full information on the approval of the study protocol must also be provided in the manuscript.

Flow Cytometry

Plots

Confirm that:

☒ The axis labels state the marker and fluorochrome used (e.g. CD4-FITC).

☒ The axis scales are clearly visible. Include numbers along axes only for bottom left plot of group (a 'group' is an analysis of identical markers).

☒ All plots are contour plots with outliers or pseudocolor plots.

☒ A numerical value for number of cells or percentage (with statistics) is provided.

Methodology

Sample preparation	colons were aseptically dissected from mice anesthetized with isoflurane, rinsed twice in ice-cold PBS, and incubated while shaking for 20 min at 37°C in HBSS buffer containing 5mM EDTA and 10mM HEPES. Following 20 s vortex, supernatant was discarded and remaining tissues were incubated while shaking for 20 min at 37°C in pre-warmed RPMI buffer containing 4% FCS, 0.5 U/ml dispase, 0.5 mg/ml collagenase D, 0.25 mg/ml DNaseI. Following 20 s vortex, supernatant was discarded and tissues were minced in pre-warmed buffer and digested while shaking for an additional 45 min at 37°C. Following 20 s vortex, digested suspension was passed through a 70 um cell strainer, washed with ice cold RPMI, and centrifuged at 2000 rpm for 10 min at 4°C. Pelleted cells were resuspended in complete RPMI<br>Briefly, anesthetized mice were transcardially perfused with PBS, intact brains were dissected, stored in Hibernate-A medium (Thermofisher), and then transferred into a dounce homogenizer containing HBSS supplemented with 10% FBS, glutamine, and pen/strep. Tissues were homogenized by passing with slide plunger 6-8 times, triturated using a 5 ml serological pipet 5-6 times, and then passed through a 70 um filter with an additional wash with buffer. Suspension was centrifuged for 5 min at 500 g, and supernatant was discarded. Cells were resuspended in 1 ml 30% percoll, inverted 4-5 times, and then centrifuged for 7 min at 500 g. Myelin layer was carefully decanted, pellet was resuspended in ice-cold PBS, and centrifuged for 5 min at 500 g. Finally, cells were resuspended in PBS
Instrument	BD LSR Fortessa flow cytometer
Software	FACSDiva

Cell population abundance	No cell sorting procedure was used in this study
Gating strategy	Cells were defined by size and granularity in FSC-A vs. SSC-A plots. Subsequently, duplets were excluded in FSC-A vs FSC-H plots and dead cells were excluded by means of fixable viability dye positivity. T cells were gated according to the lineage marker CD45 and CD3, then T cell types were gated in CD4 vs CD8 plots. Th17 cells were gated as IL-17+ population of CD4 T cells while TH1 cells were gated as IFNg+ population of CD4 T cells. Macrophages were gated as CD45 +CD11b+CD64+MHCII+ and DCs were gated as CD11C+CD103+MHCII+ population of living single cells in the colon sample. Microglia were gated as CD45lowCD11b+ living single cells in CNS samples. Gating strategies of multi- parameter flow cytometry analysis is shown in Extended Data Figures. Boundaries between positive and negative cells were defined by use of fluorescence minus one (FMO) controls.

☒ Tick this box to confirm that a figure exemplifying the gating strategy is provided in the Supplementary Information.
